# Coordinated Regulation of Myeloid-Derived Suppressor Cells by Cytokines and Chemokines

**DOI:** 10.3390/cancers14051236

**Published:** 2022-02-27

**Authors:** Ru Li, Mousumi Beto Mukherjee, Jun Lin

**Affiliations:** Department of Anesthesiology, Stony Brook University School of Medicine, Stony Brook, NY 11794, USA; ru.li@stonybrookmedicine.edu (R.L.); mousumi.betomukherjee@stonybrookmedicine.edu (M.B.M.)

**Keywords:** myeloid-derived suppressor cells, cytokines, chemokines, cancer metastasis

## Abstract

**Simple Summary:**

In this review, we summarize the effects of various cytokines and chemokines as a network to regulate the expansion, recruitment, and immunosuppressive functions of myeloid-derived suppressor cells in cancer metastasis.

**Abstract:**

Myeloid-derived suppressor cells (MDSCs) are immature myeloid cells that impair immune cell functions and promote tumor progression. Mounting evidence indicates that cytokines and chemokines in the tumor microenvironment alter MDSCs. Various cytokines and chemokines are involved in MDSC production, their infiltration into tumors, and their exertion of suppressive functions. Here, we consider those cytokines, chemokines, and MDSCs as an intricately connected, complex system and we focus on how tumors manipulate the MDSCs through various cytokines and chemokines. We also discuss treatment capitalizing on cytokines/chemokine signaling aimed at combating the potent immunosuppressive activities of MDSCs to improve disease outcomes.

## 1. Introduction

Myeloid-derived suppressor cells (MDSCs) are immature myeloid cells derived from bone marrow progenitor cells with potent suppression of antitumor immunity to support tumor progression. Elevated levels of MDSCs in the peripheral blood and tumor microenvironment (TME) have been correlated with cancer progression. MDSCs are a heterogeneous population of immune cells identified in both mice and humans, including polymorphonuclear MDSCs (PMN-MDSCs) and monocytic MDSCs (M-MDSCs). MDSCs are distinguished from neutrophils and monocytes by several biochemical and genomic characteristics. The phenotypic characteristics of different subsets of MDSC in both humans and mice are summarized in Table 1. PMN-MDSCs have increased expression of NADPH oxidase (Nox2), which increases the release of reactive oxygen species (ROS) without nitric oxide (NO) production. M-MDSCs have increased expression of nitric oxide synthase 2 (Nos2), which produce considerable levels of NO without ROS [1]. Interestingly, MDSCs in peripheral lymphoid organs and blood are mostly PMN-MDSCs with modest suppressive activity and that differentiate into macrophages and dendritic cells. MDSCs migrated into TME are dominantly M-MDSCs with enhanced suppressive phenotypes that rapidly differentiate into tumor-associated macrophages [2]. It has been suggested that exposure of MDSCs to hypoxia in TME was implicated in the regulation of MDSC differentiation, and HIF1α (Hypoxia-inducible factor 1-alpha) were found to be primarily involved in the observed effects. The nature of immune suppression by MDSCs is to hamper the recognition of tumor cells by cytotoxic T lymphocytes in non-specific ways through ROS, Arg-1, NO, and peroxynitrite (PNT), and in an antigen-specific way. In TME, MDSCs suppressed both non-specific and antigen-specific T cell activity with antigen-specific suppression predominantly. As soluble mediators, cytokines and chemokines are crucial in the immune system as well as finely tuned in tumor growth and angiogenesis during metastasis. The diverse features of cytokines and chemokines combined with their effects on MDSC results in various targets that influence cancer outcomes. However, most studies examined individual cytokines and chemokines independently, which produced fragmented information and led to a convoluted view of their role in MDSC functions in cancer. In this review, we present a comprehensive overview of individual cytokine- and chemokine-signaling targeting MDSCs in TME.

## 2. Regulation of MDSC Expansion by Cytokines and Chemokines

**Figure 1:**
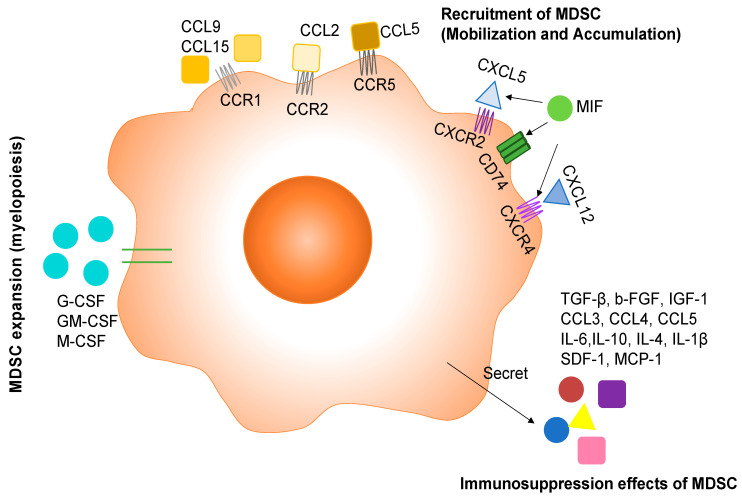
The role of cytokines and chemokines in MDSC development and function. Tumor cells mainly release G-CSF, GM-CSF, and M-CSF to induce the production of MDSC. Then the directing of MDSCs towards tumor and metastatic sites is mediated by chemokines belonging to the C-C chemokine family and C-X-C chemokine family. MDSCs exert immunosuppressive effects through secreting a variety of inflammatory cytokines and chemokines.

The myeloid progenitor cells in bone marrow and lymphatic organs undergo myelopoiesis in responding to persistent stimulation from tumors. These signals include a group of cytokines named colony-stimulating factors (CSFs). CSFs include granulocyte colony-stimulating factor (G-CSF), granulocyte/macrophage colony-stimulating factor (GM-CSF), and macrophage colony-stimulating factor (M-CSF). G-CSF and GM-CSF have overlapped functions, and signaling is mainly through JAK/STAT3/ERK/PI3K [6]. Metastatic murine 4T1 cells released G-CSF under in vitro conditions and in the 4T1 syngenetic mouse model, which initiated the premetastatic environment in lungs by attracting MDSCs. The infiltration of MDSC in lungs induced angiogenesis and enhanced the metastatic ability of cancer cells [7]. Another study identified GM-CSF, M-CSF, CCL2 (C-C motif chemokine ligand 2), and TNFα (tumor necrosis factor α) released by invasive squamous cells with the loss of p120-catenin in TME. The accompanying TME was found to be filled with MDSCs [8]. Chemotherapy significantly enhanced the production of GM-CSF from various PDAC (pancreatic ductal adenocarcinoma cell) cell lines and PDAC tumor tissues from patients. GM-CSF further induced the differentiation of monocytes into MDSC in vitro [9]. In glioblastoma, tumor cells and brain damage induced by growing tumor would synthesize G-CSF and GM-CSF to stimulate myelopoiesis in bone marrow [10] (Figure 1).

## 3. Recruitment of MDSCs to TME

Primary tumors, metastatic tumors, and tumor-associated stromal cells all secrete various cytokines, chemokines, and transcription factors to recruit MDSCs into TME. Different tumors have no preference in producing cytokines, chemokines, and transcription factors. All these soluble factors get into peripheral blood, bone marrow, and lymphatic organs (lymph node and spleen) to guide MDSCs from the bone marrow and lymphatic organs to the tumor, which includes mobilizing MDSCs into the blood and accumulating MDSCs from the blood to the tumors. This process is primarily regulated by CCL2 for M-MDSCs and CCL5 for PMN-MDSCs. Ubiquitous expression of CCL2 and its receptor CCR2 has been identified in patient samples from various types of cancers [11,12]. Specifically, MDSCs expressed many chemokine receptors, including CCR2 [13]. The CCL2/CCR2 signaling pathway was implicated in the migration of MDSC to tumor in vitro and in vivo. Qian et al. showed that tumor and stromal-derived CCL2 recruited MDSCs (Gr1+ inflammatory monocytes referred to in the study) preferentially to pulmonary metastasis but not the primary tumor in the polyoma middle T (PyMT) model of breast cancer [14]. In a mouse model of gliomas, microglia and macrophages in TME secreted CCL2 to recruit regulatory T cells and MDSCs [15]. A recent study analyzing periodontal inflammation with a 4T1 syngenetic mouse model found that the production of CCL2 as well CCL5, CXCL12, and CXCL5 was regulated by IL-1β signaling. These chemokines recruited MDSC in cervical lymph nodes to establish the premetastatic niche, and finally the mice with periodontal inflammation developed more lymph node micrometastasis compared with the control group [16]. CCL5 binds to CCR5 and has been shown to direct the mobilization of PMN-MDSCs from bone marrow to blood for tumor progression [17]. In addition, CCR1+ MDSC has also been identified in metastatic cancer. In a mouse model of colorectal cancer liver metastasis, colorectal cancer cells recruited CCR1^+^ myeloid cells (mostly granulocytic MDSC phenotype) by expressing CCL-9 to expand metastatic foci in the liver [18], and the enriched myeloid cells in metastatic foci included CCR1^+^ neutrophils, eosinophils monocytes, and fibrocytes [19]. Furthermore, the same group showed that colorectal cancer cells with the loss of the tumor suppressor SMAD4 secreted CCL15 and recruited CCR1^+^ myeloid cells to assist tumor invasion in colorectal cancer patients [20]. (Figure 1)

Another important group of chemokines in fostering MDSC accumulation in TME is C-X-C motif chemokine ligands, including CXCL5 and CXCL12. CXCL5 binds to C-X-C motif chemokine receptor 2 (CXCR2), and CXCL12 binds to CXCR4. The CXCL5/CXCR2 axis was involved in recruiting MDSCs into mammary carcinomas with type II TGFβ receptor gene deletion in the PyMT model [21]. In a spontaneous murine model of melanoma, CXCL5 attracted PMN-MDSCs to primary tumor to assist cancer cell dissemination by inducing epithelial–mesenchymal transition (EMT) [22]. Loss of CXCR2 dramatically suppresses colonic chronic inflammation and colitis-associated tumorigenesis through inhibiting infiltration of myeloid-derived suppressor cells (MDSCs) into colonic mucosa and tumors in a mouse model of colitis-associated cancer [23]. In ovarian cancer patients, tumor-associated inflammatory mediator prostaglandin E_2_ (PGE2) induced the production of CXCL12(SDF1) in TME and functional expression of CXCR4 on MDSCs, which further attracted MDSCs into the TME [24]. The CXCL12/CXCR4 axis also participated in the MDSC recruitment in TME of MDSCs into mammary carcinomas with type II TGF-β receptor gene as described above [21]. (Figure 1)

Additional active molecules as potent chemoattractants for MDSCs emanate from studies showing that pro-inflammatory proteins S100A8 and S100A9 as the downstream targets of STAT3, a transcription factor regulating cancer cell proliferation and survival, were implicated in an autocrine pathway for the accumulation of MDSC in tumors and metastatic sites [25,26,27]. In addition, tumor cells induced the expression of MIF expression to increase monocytic MDSCs within the tumor, which in turn promoted tumor growth and metastasis in a 4T1 syngenetic mouse model [28]. Under an in vitro condition, MIF promoted the differentiation of CD11b^+^ cells into monocytic MDSCs [28].

## 4. Immunosuppression Effects of MDSCs

MDSCs employ a variety of mechanisms to inhibit the anti-tumor activity of immune cells, thereby supporting tumor growth and metastasis. One of immunomodulatory functions of MDSCs is the activation and expansion of Treg cells. In peripheral lymphoid organs, MDSCs release TGF-β to induce differentiation and/or proliferation of Foxp3^+^ Tregs and block NK cell activity [29,30]. Tumor-infiltrating MDSCs could also recruit CCR5+ Tregs to the tumor site through releasing CCR5 ligands CCL3, CCL4, and CCL5 to promote tumor growth in a B16 melanoma model [31]. In addition to the immunosuppressive effect, MDSCs could also promote tumor progression through affecting cancer cells directly. MDSCs in TME released IL-6 to constantly activate STAT3 in cancer cells, which conferred invasive potential to cancer cells [32], induced epithelial mesenchymal transition (EMT) of cancer cells [33], and enhanced cancer cell stemness [34]. MDSCs also promoted distant metastases by establishing the pre-metastatic niche. As regulated by the primary tumor, MDSCs arrived in the metastatic organs before the tumor cells and conditioned it to promote tumor seeding by secreting b-FGF, IGF-1, IL-10, IL-4, IL-1β, SDF-1, and MCP-1 [35]. IL-17 could also be involved in the immune-suppressive function of MDSC in the mammary carcinoma model. IL-17 upregulated the expression of Arg-1, MMP-9, indoleamine 2,3-dioxygenase (IDO), COX-2, and MMP13 in MDSCs to increase their immune-suppressive function [36]. Transmembrane, but not the secreted TNF-α, enhanced the suppressive activity of MDSCs through up-regulating Arg-1 and inducible NO synthase (iNOS), promoting the release of NO, ROS, IL-10, and TGF-β [37] (Figure 1).

## 5. Cytokines/Chemokines Regulating Neutrophils and Monocytes in Cancer

It remains disputed whether MDSCs compared with neutrophils and monocytes are different cell types or cellular states. Neutrophils and PMN-MDSCs share an origin and many morphological and phenotypic features, but they have different biological functions, which is also applied to monocyte and M-MDSC. PMN-MDSC and M-MDSC have been indicated as pathologically activated neutrophils and monocytes, respectively [4]. Moreover, MDSCs take the same differentiation pathways as neutrophils and monocytes. Monocytes and M-MDSC can be distinguished based on expression of MHC class II molecules. On the other hand, the distinction between PMN-MDSC and tumor-associated neutrophils is more difficult. MDSC and tumor-promoting neutrophils clearly differentiate into divergent cell types, as MDSCs have reduced expression of CD16 and CD62L and increased expression of Arg-1, CD66B, and CD11b [38,39]. In appreciation of this complexity, to extend our understanding of cytokines and chemokines in immunosuppressive TME, we discuss the cytokines and chemokines involved in the interaction between neutrophiles, monocytes, and cancer cells in tumor metastasis.

### 5.1. Neutrophils

Neutrophils are the most abundant leukocytes in the blood and the primary responsive immune cells against inflammation and infection. Recent studies have shown that neutrophils are a predominant component of the TME and are involved in both anti-tumor and pro-tumor activities [40]. Neutrophils at the tumor site secrete various cytokines and chemokines to enhance their own recruitment towards TME along with the enrolment, activation, and polarization of other immune cells. Neutrophils recruited via cytokines and chemokines to cancer cells can have both anticancer and tumor-promoting effects, either of which may be most likely depending on the type of neutrophils and/or factors present in the TME. Based on their functions, neutrophils are classified into tumor-suppressing (N1) and tumor-promoting (N2), similar to M1 and M2 tumor-associated macrophages (TAM) [41]. Type I interferons (IFN-I) and TGF-β are major factors involved in the polarization of neutrophils.

#### 5.1.1. The Anti-Tumor Role of Neutrophils

**Figure 2:**
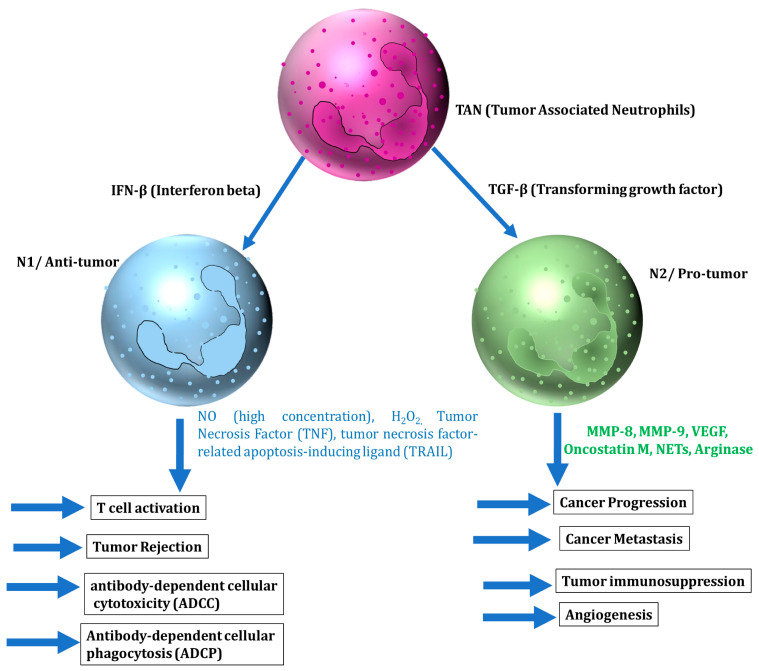
The role of cytokines and chemokines relevant to neutrophils in affecting tumor progression.

Neutrophils are cytotoxic to tumor cells both in vitro and in vivo. Anti-tumor activity of neutrophils is regulated via various factors, such as the release of nitric oxide induced by hepatocyte growth factor (HGF) through binding to tyrosine-protein kinase MET, the generation of hydrogen peroxide (H_2_O_2_), and the secretion of the tumor necrosis factor TNF-α [42,43]. The ability of neutrophil-secreted cytokines to produce anticancer activity has been widely studied. The N1 phenotype with high levels of TNF-α, IL-2, IL-4, IL-7, and IL-10 expression induces a cytotoxic effect to cancer cells and hampers cancer cell progression [44,45]. TNF-α particularly, as a major cytokine from the N1 neutrophil, enhances the transmigration of neutrophil and released nitric oxide (NO), which induced apoptosis in cancer cells and inhibited cancer growth [45] (Figure 2).

#### 5.1.2. The Tumor-Promoting Role of Neutrophils

N2 neutrophils have strong immunosuppressive and tumor-promoting activities, including the promotion of tumor angiogenesis, invasion, and metastases via various cytokines and chemokines [40]. The interplay between N2 neutrophils and cytokines in the TME has an important role in tumor metastasis. It has been shown that brain metastatic cells upregulated the pro-tumorigenic cytokine G-CSF to attract the immunosuppressive N2 neutrophils into the brain and facilitate brain metastasis [46]. G-CSF and IL-6 produced by tumor cells in circulating conditions also induced the accumulation of neutrophils in pre-metastatic organs to facilitate the colonization of disseminated cancer cells [47]. Inflammatory cytokine IL-6 and CCL3 were observed to recruit neutrophils to aid in breast cancer lung metastasis [48]. Other inflammatory cytokines such as IL-8 and IL-1β triggered the formation of neutrophil extracellular traps (NETs), which degraded thrombospondin-1 and supported the metastatic growth of cancer cells in the lungs [48,49]. IL-1β also induced IL-17 expression from γδ T cells, which then regulated neutrophil expansion and polarization via induction of G-CSF [50]. All these inflammatory cytokines cooperate within a network of systemic inflammation to facilitate metastatic formation (Figure 2).

### 5.2. Monocytes

Monocytes are the third most abundant form of immune cells in peripheral blood. Inflammatory monocytes (CCR2+Ly6C^hi^) are an important population infiltrating into primary and metastatic tumor sites for cancer progression, especially through their interaction with the endothelium to affect angiogenesis and vascular permeability. Angiopoietin-2, as an important regulator of blood vessel stabilization and angiogenesis, has been shown to recruit the Tie-2+ monocyte to hypoxic tumor areas, leading to their differentiation into macrophages [51]. The second important chemoattractant is CCL2, which was secreted by mammary tumors and which recruited CCR2-expressing inflammatory monocytes to pulmonary metastatic sites of breast cancer. Those inflammatory monocytes produced VEGF to facilitate tumor cell extravasation [14]. In another study, the infiltrated inflammatory monocytes induced blood vessel formation, cancer cell proliferation in metastasis, and IL-6 production in mice [52]. In a 4T1 syngenetic mouse model of breast cancer metastasis, IL-1β as a master cytokine promoted the recruitment of CCR2+ monocytes and differentiation to inflammatory monocytes [53]. In human hepatocellular carcinoma, most abundantly expressed CCL15 recruited CCR1+ CD14+ monocytes toward HCC invasive margin to enhance tumor metastasis by inducing immune suppression and angiogenesis [54]. Besides inflammatory monocytes, there is another subpopulation of monocyte named patrolling monocyte (Ly6C^lo^). Patrolling monocytes are largely localized in the circulation and may have antitumor functions. In murine models of Lewis lung carcinoma, melanoma, and mammary tumor, patrolling monocytes could prevent lung metastasis by interacting with metastatic tumor cells, scavenging tumor debris in the TME, and recruiting anti-tumoral natural killer cells locally [55].

## 6. Therapies Targeting MDSCs in Cancer Treatment

Targeting immune-suppressive cells should be an intricate combination of selective inhibition of cytokines or depletion of MDSCs alongside bolstering anti-tumor immunity. A variety of strategies have been applied over the past decade to evaluate the safety and efficacy of MDSC inhibition to prevent cancer progression (Table 2). The first approach is to deactivate MDSCs’ immunosuppressive functions on T cell activity, such as by inhibiting certain molecular mechanisms, including targeting Arg-1, iNOS, and STAT3 activities. Some immunosuppressive functions are shared among all subtypes of myeloid cells, but some are unique to certain populations. Targeting common mechanisms is likely to be more effective than targeting individual suppressive pathways. One example is Sunitinib, a small-molecule, synthetic indoline-based receptor tyrosine kinase (RTK) inhibitor. In patients with oligometastases of various cancer types, sunitinib treatment decreased the suppressive function of M-MDSCs by reducing their expression of Arg-1 and phosphorylated STAT3, increased T-cell proliferative activity, and improved progression-free survival [56]. In patients with high-risk non-muscle invasive bladder cancer, sunitinib resolved the MDSC-mediated immunosuppression, but did not improve the clinical outcomes [57]. Another class of drugs targeting MDSC is phosphodiesterase type 5 (PDE5) inhibitors, such as Tadalafil. PDE5 inhibitors have been shown to decrease the expression of IL-4Rα, which reduced phosphorylation of STAT6 and Arg-1 expression to effectively block MDSC functions [58,59]. In patients with head and neck cancer, two clinical trials demonstrated that tadalafil treatment effectively reduced MDSC and Treg and increased tumor-specific CD8+ T cells [60,61].

MDSC depletion is another avenue for cancer therapeutics which correlates with diminished tumor growth. There have been some successes in triggering MDSC apoptosis with the off-target effects of conventional systemic chemotherapeutic agents such as gemcitabine and 5-fluorouracil. Both gemcitabine and 5-fluorouracil reduced the numbers and effectiveness of MDSCs in the TME using animal models [64,65]. In clinical practice, gemcitabine and 5-fluorouracil are often used in combination with other drugs, and they have been shown to decrease the density of MDSC, leading to the restoration of immune functions and tumor regression [63,66,67]. It is still not clear whether the systemic neutralization of MDSC or the targeting of tumor-infiltrating MDSC is the best option. The answer would be important to guide the future therapeutic strategy.

There are also therapies targeting MDSCs trafficking from bone marrow to peripheral lymph nodes and tumor sites via different molecules, as described above. There are several drugs that have been developed against inflammatory cytokines, including a human monoclonal antibody carlumab for CCL2 [68], a selective CCR2 antagonist (PF-04136309) [69], a CCR5 antagonist (Maraviroc) [70], and a small-molecule CSF-1R inhibitor (Pexidartinib) [71]. Most clinical trials have examined the safety and efficacy of those drugs in various cancer models. Although they were studied in animal models to reduce MDSCs, none of them has yet been evaluated on MDSC accumulation in patients. It has to be noted that blocking one specific cytokine may lead to compensation by a redundant cytokine, since there are many functional overlaps between different cytokines, which may neutralize the beneficial effects. Thus, a combination of different drugs should be considered in future clinical trials as better therapeutic approaches.

## 7. Conclusions

MDSCs are generated under chronic pathological conditions such as cancer. As one of the most potent immunosuppressive cells, MDSCs promote tumor progression by inhibition T and NK cells as well as direct effect on neovascularization and tumor cell invasion. Therefore, MDSC represent a crucial target in cancer treatment. Here we reviewed the cytokine/chemokine signals for MDSC survival, differentiation, and maintenance. Understanding the mechanisms and key regulators of MDSC development is critically important to overcoming immunosuppression and can be translated to better therapeutic targets.

## Figures and Tables

**Table 1:** Phenotypic characteristics of biomarkers for identification of cells as MDSC.

Types of MDSCs	Human	Mice
PMN-MDSC	CD11b^+^CD14^−^CD15^+^, CD11b^+^CD14^−^CD66b^+^ [3] LIN^−^CD11b^+^HLA-DR^−^CD33^+^, LIN^−^CD11b^+^HLA-DR^−^CD66b^+^, LIN^−^CD11b^+^HLA-DR^−^CD15^+^, LIN^−^CD11b^+^HLA-DR^−^CD15^+^CD66b^+^ [4] CD11b^+^CD14^-^CD33^+^ (additionally CD15 and/or CD66b) [4] CD11b^+^HLA-DR^−^CD15^+^CD14^−^CD33^mid^ [5]	CD11b (+)Ly6G(+)Ly6C(low) [3,5] CD11b^+^GR1^hi^LY6C^low^LY6G^+^CD49d^−^ [4]
M-MDSC	CD11b^+^CD14^+^HLA-DR^−/low^CD15^−^ [3] CD33^+^CD14^+^HLA-DR^low/−^ [4] CD11b^+^HLA-DR^−^CD15^−^CD14^+^CD33^hi^ [5]	CD11b(+)Ly6G(−)Ly6C (high) [3,5] CD11b^+^GR1^mid^LY6C^hi^LY6G^−^CD49d^+^ [4]

**Table 2:** Therapeutic strategies targeting MDSCs in cancer.

Strategy	Drugs/Chemokines	Mechanism
Inducing MDSC Differentiation	Inhibiting the molecular mechanisms targeting Arg-1, iNOS, and STAT3 activities	Deactivating MDSC immunosuppressive functions on T cell activity.
Targeting MDSC activity	Sunitinib (a small-molecule, synthetic indoline-based receptor tyrosine kinase (RTK) inhibitor)	Decreasing the suppressive function of M-MDSCs by reducing their expression of Arg-1 and phosphorylated STAT3/increasing T cell proliferative activity/improving progression-free survival/in high-risk non-muscle invasive bladder cancer, sunitinib can resolve MDSC-mediated immunosuppression.
	Tadalafil (phosphodiesterase type 5 (PDE5) inhibitor)	Decreasing the expression of IL-4Rα, resulting in the reduction of the phosphorylation of STAT6 and Arg-1 expression to effectively block MDSC functions/a clinical trial has proven the reduction of MDSC and Treg/increasing tumor-specific CD8+ T cells.
MDSC depletion	Using systemic chemotherapeutic agents such as gemcitabine, 5-fluorouracil, paclitaxel [62], and gemcitabine [63]	Reducing the numbers and effectiveness of MDSCs in the TME using animal models/in combination with other drugs, has shown decreased density of MDSC, leading to the restoration of immune functions and tumor regression/low-dosage chemotherapeutic treatment with paclitaxel, gemcitabine was found to deplete MDSC populations and improve anti-tumor immune action in mice model.
Blocking MDSC recruitment and trafficking	Drugs developed against inflammatory cytokines, including a human monoclonal antibody carlumab for CCL2, a selective CCR2 antagonist (PF-04136309), a CCR5 antagonist (Maraviroc), and a small-molecule CSF-1R inhibitor (Pexidartinib)	Effectively blocking the chemokine receptors present on MDSCs due to ligand redundancy, as these chemokines play an important role in the recruitment of MDSCs.

## Abbreviations

Arg-1	arginase 1
CSFs	colony-stimulating factors
CCL2	C-C motif chemokine ligand 2
CCL5	C-C motif ligand 5
CCR2	C-C chemokine receptor type 2
CXCR2	C-X-C motif chemokine receptor 2
EMT	epithelial–mesenchymal transition
G-CSF	granulocyte colony-stimulating factor
GM-CSF	granulocyte/macrophage colony-stimulating factor
HIF1α	hypoxia-inducible factor 1-alpha
HGF	hepatocyte growth factor
IL-1β	interleukin-1beta
IL-6	interleukin-6
iNOS	inducible NO synthase
IFN-I	type I interferons
IL-4Rα	interleukin-4 receptor alpha
MDSCs	myeloid-derived suppressor cells
M-MDSCs	monocytic MDSCs
M-CSF	macrophage colony-stimulating factor
MMP	matrix metalloproteinase
Nox2	NADPH oxidase
NO	nitric oxide
Nos2	nitric oxide synthase 2
N1	tumor-suppressing neutrophils
N2	tumor-promoting neutrophils
NETs	neutrophil extracellular traps
PMN-MDSCs	polymorphonuclear MDSCs
PNT	peroxynitrite
PDAC	pancreatic ductal adenocarcinoma cell
PyMT	polyoma Middle T
PGE2	prostaglandin E2
PDE5	phosphodiesterase type 5
TNFα	tumor necrosis factor α
TME	tumor microenvironment
TGFβ	transforming growth factor beta
TAM	tumor-associated macrophages
ROS	reactive oxygen species
RTK	receptor tyrosine kinases
STAT3	signal transducer and activator of transcription 3

## References

[1] Youn J.-I., Nagaraj S., Collazo M., Gabrilovich D.I. (2008). Subsets of Myeloid-Derived Suppressor Cells in Tumor-Bearing Mice. J. Immunol..

[2] Kumar V., Patel S., Tcyganov E., Gabrilovich D.I. (2016). The Nature of Myeloid-Derived Suppressor Cells in the Tumor Microenvironment. Trends Immunol..

[3] Bronte V., Brandau S., Chen S.-H., Colombo M.P., Frey A.B., Greten T.F., Mandruzzato S., Murray P.J., Ochoa A., Ostrand-Rosenberg S. (2016). Recommendations for myeloid-derived suppressor cell nomenclature and characterization standards. Nat. Commun..

[4] Gabrilovich D.I., Ostrand-Rosenberg S., Bronte V. (2012). Coordinated regulation of myeloid cells by tumours. Nat. Rev. Immunol..

[5] Cassetta L., Baekkevold E.S., Brandau S., Bujko A., Cassatella M.A., Dorhoi A., Krieg C., Lin A., Loré K., Marini O. (2019). Deciphering myeloid-derived suppressor cells: Isolation and markers in humans, mice and non-human primates. Cancer Immunol. Immunother..

[6] Mehta H.M., Malandra M., Corey S.J. (2015). G-CSF and GM-CSF in Neutropenia. J. Immunol..

[7] Kowanetz M., Wu X., Lee J., Tan M., Hagenbeek T.J., Qu X., Yu L., Ross J., Korsisaari N., Cao T. (2010). Granulocyte-colony stimulating factor promotes lung metastasis through mobilization of Ly6G+Ly6C+ granulocytes. Proc. Natl. Acad. Sci. USA.

[8] Stairs D.B., Bayne L.J., Rhoades B., Vega M.E., Waldron T.J., Kalabis J., Klein-Szanto A., Lee J.-S., Katz J.P., Diehl J.A. (2011). Deletion of p120-Catenin Results in a Tumor Microenvironment with Inflammation and Cancer that Establishes It as a Tumor Suppressor Gene. Cancer Cell.

[9] Takeuchi S., Baghdadi M., Tsuchikawa T., Wada H., Nakamura T., Abe H., Nakanishi S., Usui Y., Higuchi K., Takahashi M. (2015). Chemotherapy-Derived Inflammatory Responses Accelerate the Formation of Immunosuppressive Myeloid Cells in the Tissue Microenvironment of Human Pancreatic Cancer. Cancer Res..

[10] Kast R.E., Hill Q., Wion D., Mellstedt H., Focosi D., Karpel-Massler G., Heiland T., Halatsch M.-E. (2017). Glioblastoma-synthesized G-CSF and GM-CSF contribute to growth and immunosuppression: Potential therapeutic benefit from dapsone, fenofibrate, and ribavirin. Tumor Biol..

[11] Huang B., Lei Z., Zhao J., Gong W., Liu J., Chen Z., Liu Y., Li D., Yuan Y., Zhang G.-M. (2007). CCL2/CCR2 pathway mediates recruitment of myeloid suppressor cells to cancers. Cancer Lett..

[12] Chun E., Lavoie S., Michaud M., Gallini C.A., Kim J., Soucy G., Odze R., Glickman J.N., Garrett W.S. (2015). CCL2 Promotes Colorectal Carcinogenesis by Enhancing Polymorphonuclear Myeloid-Derived Suppressor Cell Population and Function. Cell Rep..

[13] Ben-Meir K., Twaik N., Baniyash M. (2018). Plasticity and biological diversity of myeloid derived suppressor cells. Curr. Opin. Immunol..

[14] Qian B.-Z., Li J., Zhang H., Kitamura T., Zhang J., Campion L.R., Kaiser E.A., Snyder L.A., Pollard J.W. (2011). CCL2 recruits inflammatory monocytes to facilitate breast-tumour metastasis. Nature.

[15] Chang A.L., Miska J., Wainwright D.A., Dey M., Rivetta C.V., Yu D., Kanojia D., Pituch K.C., Qiao J., Pytel P. (2016). CCL2 Produced by the Glioma Microenvironment Is Essential for the Recruitment of Regulatory T Cells and Myeloid-Derived Suppressor Cells. Cancer Res..

[16] Cheng R., Billet S., Liu C., Haldar S., Choudhury D., Tripathi M., Hav M., Merchant A., Hu T., Huang H. (2020). Periodontal inflammation recruits distant metastatic breast cancer cells by increasing myeloid-derived suppressor cells. Oncogene.

[17] Hawila E., Razon H., Wildbaum G., Blattner C., Sapir Y., Shaked Y., Umansky V., Karin N. (2017). CCR5 Directs the Mobilization of CD11b+Gr1+Ly6Clow Polymorphonuclear Myeloid Cells from the Bone Marrow to the Blood to Support Tumor Development. Cell Rep..

[18] Kitamura T., Fujishita T., Loetscher P., Revesz L., Hashida H., Kondoh S., Aoki M., Taketo M.M. (2010). Inactivation of chemokine (C-C motif) receptor 1 (CCR1) suppresses colon cancer liver metastasis by blocking accumulation of immature myeloid cells in a mouse model. Proc. Natl. Acad. Sci. USA.

[19] HideyoHirai H., Fujishita T., Kurimoto K., Miyachi H., Kitano S., Inamoto S., Itatani Y., Saitou M., Maekawa T., Taketo M.M. (2014). CCR1-mediated accumulation of myeloid cells in the liver microenvironment promoting mouse colon cancer metastasis. Clin. Exp. Metastasis.

[20] Inamoto S., Itatani Y., Yamamoto T., Minamiguchi S., Hirai H., Iwamoto M., Hasegawa S., Taketo M.M., Sakai Y., Kawada K. (2016). Loss of SMAD4 Promotes Colorectal Cancer Progression by Accumulation of Myeloid-Derived Suppressor Cells through the CCL15–CCR1 Chemokine Axis. Clin. Cancer Res..

[21] Yang L., Huang J., Ren X., Gorska A.E., Chytil A., Aakre M., Carbone D.P., Matrisian L.M., Richmond A., Lin P.C. (2008). Abrogation of TGFβ Signaling in Mammary Carcinomas Recruits Gr-1+CD11b+ Myeloid Cells that Promote Metastasis. Cancer Cell.

[22] Toh B., Wang X., Keeble J., Sim W.J., Khoo K., Wong W.-C., Kato M., Prevost-Blondel A., Thiery J.-P., Abastado J.-P. (2011). Mesenchymal Transition and Dissemination of Cancer Cells Is Driven by Myeloid-Derived Suppressor Cells Infiltrating the Primary Tumor. PLOS Biol..

[23] Katoh H., Wang D., Daikoku T., Sun H., Dey S.K., Dubois R.N. (2013). CXCR2-Expressing Myeloid-Derived Suppressor Cells Are Essential to Promote Colitis-Associated Tumorigenesis. Cancer Cell.

[24] Obermajer N., Muthuswamy R., Odunsi K.O., Edwards R.P., Kalinski P. (2011). PGE2-Induced CXCL12 Production and CXCR4 Expression Controls the Accumulation of Human MDSCs in Ovarian Cancer Environment. Cancer Res..

[25] Hiratsuka S., Watanabe A., Aburatani H., Maru Y. (2006). Tumour-mediated upregulation of chemoattractants and recruitment of myeloid cells predetermines lung metastasis. Nat. Cell Biol..

[26] Sinha P., Okoro C., Foell D., Freeze H., Ostrand-Rosenberg S., Srikrishna G. (2008). Proinflammatory S100 Proteins Regulate the Accumulation of Myeloid-Derived Suppressor Cells. J. Immunol..

[27] Ichikawa M., Williams R., Wang L., Vogl T., Srikrishna G. (2011). S100A8/A9 Activate Key Genes and Pathways in Colon Tumor Progression. Mol. Cancer Res..

[28] Simpson K.D., Templeton D.J., Cross J.V. (2012). Macrophage Migration Inhibitory Factor Promotes Tumor Growth and Metastasis by Inducing Myeloid-Derived Suppressor Cells in the Tumor Microenvironment. J. Immunol..

[29] Huang B., Pan P.-Y., Li Q., Sato A.I., Levy D., Bromberg J., Divino C.M., Chen S.-H. (2006). Gr-1+CD115+ Immature Myeloid Suppressor Cells Mediate the Development of Tumor-Induced T Regulatory Cells and T-Cell Anergy in Tumor-Bearing Host. Cancer Res..

[30] Mao Y., Sarhan D., Steven A., Seliger B., Kiessling R., Lundqvist A. (2014). Inhibition of Tumor-Derived Prostaglandin-E2 Blocks the Induction of Myeloid-Derived Suppressor Cells and Recovers Natural Killer Cell Activity. Clin. Cancer Res..

[31] Schlecker E., Stojanovic A., Eisen C., Quack C., Falk C.S., Umansky V., Cerwenka A., Xu M., Hadinoto V., Appanna R. (2012). Tumor-Infiltrating Monocytic Myeloid-Derived Suppressor Cells Mediate CCR5-Dependent Recruitment of Regulatory T Cells Favoring Tumor Growth. J. Immunol..

[32] Oh K., Lee O.-Y., Shon S.Y., Nam O., Ryu P.D., Seo M.W., Lee D.-S. (2013). A mutual activation loop between breast cancer cells and myeloid-derived suppressor cells facilitates spontaneous metastasis through IL-6 trans-signaling in a murine model. Breast Cancer Res..

[33] Zhu H., Gu Y., Xue Y., Yuan M., Cao X., Liu Q. (2017). CXCR2+ MDSCs promote breast cancer progression by inducing EMT and activated T cell exhaustion. Oncotarget.

[34] Xu Z., Li L., Qian Y., Song Y., Qin L., Duan Y., Wang M., Li P., Jiang B., Ma C. (2019). Upregulation of IL-6 in CUL4B-deficient myeloid-derived suppressive cells increases the aggressiveness of cancer cells. Oncogene.

[35] Yan H.H., Pickup M., Pang Y., Gorska A.E., Li Z., Chytil A., Geng Y., Gray J.W., Moses H.L., Yang L. (2010). Gr-1+CD11b+ Myeloid Cells Tip the Balance of Immune Protection to Tumor Promotion in the Premetastatic Lung. Cancer Res..

[36] Novitskiy S.V., Pickup M.W., Gorska A.E., Owens P., Chytil A., Aakre M., Wu H., Shyr Y., Moses H.L. (2011). TGF-β Receptor II Loss Promotes Mammary Carcinoma Progression by Th17-Dependent Mechanisms. Cancer Discov..

[37] Hu X., Li B., Li X., Zhao X., Wan L., Lin G., Yu M., Wang J., Jiang X., Feng W. (2014). Transmembrane TNF-α Promotes Suppressive Activities of Myeloid-Derived Suppressor Cells via TNFR2. J. Immunol..

[38] Rodriguez P.C., Ernstoff M.S., Hernandez C., Atkins M., Zabaleta J., Sierra R., Ochoa A.C. (2009). Arginase I–Producing Myeloid-Derived Suppressor Cells in Renal Cell Carcinoma Are a Subpopulation of Activated Granulocytes. Cancer Res..

[39] Youn J.-I., Collazo M., Shalova I.N., Biswas S.K., Gabrilovich D.I. (2012). Characterization of the nature of granulocytic myeloid-derived suppressor cells in tumor-bearing mice. J. Leukoc. Biol..

[40] Masucci M.T., Minopoli M., Carriero M.V. (2019). Tumor Associated Neutrophils. Their Role in Tumorigenesis, Metastasis, Prognosis and Therapy. Front. Oncol..

[41] Fridlender Z.G., Sun J., Kim S., Kapoor V., Cheng G., Ling L., Scott Worthen G., Albelda S.M. (2009). Polarization of tumor-associated neutrophil pheno-type by TGF-beta: “N1” versus “N2” TAN. Cancer Cell.

[42] Finisguerra V., Di Conza G., Di Matteo M., Serneels J., Costa S., Thompson A.A.R., Wauters E., Walmsley S., Prenen H., Granot Z. (2015). MET is required for the recruitment of anti-tumoural neutrophils. Nature.

[43] Granot Z., Henke E., Comen E.A., King T.A., Norton L., Benezra R. (2011). Tumor entrained neutrophils inhibit seeding in the premetastatic lung. Cancer Cell.

[44] Musiani P., Allione A., Modica A., Lollini P.L., Giovarelli M., Cavallo F., Belardelli F., Forni G., Modesti A. (1996). Role of neutrophils and lymphocytes in inhibition of a mouse mammary adenocarcinoma engineered to release IL-2, IL-4, IL-7, IL-10, IFN-alpha, IFN-gamma, and TNF-alpha. Lab. Investig. J. Tech. Methods Pathol..

[45] Comen E., Wojnarowicz P., Seshan V.E., Shah R., Coker C., Norton L., Benezra R. (2016). TNF is a key cytokine mediating neutrophil cytotoxic activity in breast cancer patients. NPJ Breast Cancer.

[46] Zhang L., Yao J., Wei Y., Zhou Z., Li P., Qu J., Badu-Nkansah A., Yuan X., Huang Y.-W., Fukumura K. (2020). Blocking immunosuppressive neutrophils deters pY696-EZH2–driven brain metastases. Sci. Transl. Med..

[47] Li Y.-C., Zou J.-M., Luo C., Shu Y., Luo J., Qin J., Wang Y., Li D., Wang S.-S., Chi G. (2017). Circulating tumor cells promote the metastatic colonization of disseminated carcinoma cells by inducing systemic inflammation. Oncotarget.

[48] Xiao Y., Cong M., Li J., He D., Wu Q., Tian P., Wang Y., Yang S., Liang C., Liang Y. (2021). Cathepsin C promotes breast cancer lung metastasis by modulating neutrophil infiltration and neutrophil extracellular trap formation. Cancer Cell.

[49] Li B., Liu Y., Hu T., Zhang Y., Zhang C., Li T., Wang C., Dong Z., Novakovic V.A., Hu T. (2019). Neutrophil extracellular traps enhance procoagulant activity in patients with oral squamous cell carcinoma. J. Cancer Res. Clin. Oncol..

[50] Coffelt S.B., Kersten K., Doornebal C.W., Weiden J., Vrijland K., Hau C.-S., Verstegen N.J.M., Ciampricotti M., Hawinkels L.J.A.C., Jonkers J. (2015). IL-17-producing γδ T cells and neutrophils conspire to promote breast cancer metastasis. Nature.

[51] Murdoch C., Tazzyman S., Webster S., Lewis C.E. (2007). Expression of Tie-2 by Human Monocytes and Their Responses to Angiopoietin-2. J. Immunol..

[52] Bonapace L., Coissieux M.-M., Wyckoff J., Mertz K.D., Varga Z., Junt T., Bentires-Alj M. (2014). Cessation of CCL2 inhibition accelerates breast cancer metastasis by promoting angiogenesis. Nature.

[53] Kaplanov I., Carmi Y., Kornetsky R., Shemesh A., Shurin G.V., Shurin M.R., Dinarello C.A., Voronov E., Apte R.N. (2019). Blocking IL-1β reverses the immunosuppression in mouse breast cancer and synergizes with anti–PD-1 for tumor abrogation. Proc. Natl. Acad. Sci. USA.

[54] Liu L.-Z., Zhang Z., Zheng B.-H., Shi Y., Duan M., Ma L.-J., Wang Z.-C., Dong L.-Q., Dong P.-P., Shi J.-Y. (2019). CCL15 Recruits Suppressive Monocytes to Facilitate Immune Escape and Disease Progression in Hepatocellular Carcinoma. Hepatology.

[55] Hanna R.N., Cekic C., Sag D., Tacke R., Thomas G.D., Nowyhed H., Herrley E., Rasquinha N., McArdle S., Wu R. (2015). Patrolling monocytes control tumor metastasis to the lung. Science.

[56] Chen H.-M., Max S., Gildener-Leapman N., Eisenstein S., Coakley B.A., Ozao J., Mandeli J., Divino C., Schwartz M., Sung M. (2015). Myeloid-Derived Suppressor Cells as an Immune Parameter in Patients with Concurrent Sunitinib and Stereotactic Body Radiotherapy. Clin. Cancer Res. Off. J. Am. Assoc. Cancer Res..

[57] Zahoor H., Mir M.C., Barata P.C., Stephenson A.J., Campbell S.C., Fergany A., Dreicer R., Garcia J.A. (2019). Phase II trial of continuous treatment with sunitinib in patients with high-risk (BCG-refractory) non-muscle invasive bladder cancer. Investig. New Drugs.

[58] Serafini P., Meckel K., Kelso M., Noonan K., Califano J., Koch W., Dolcetti L., Bronte V., Borrello I. (2006). Phosphodiesterase-5 inhibition augments endogenous antitumor immunity by reducing myeloid-derived suppressor cell function. J. Exp. Med..

[59] Noonan K.A., Ghosh N., Rudraraju L., Bui M., Borrello I. (2014). Targeting Immune Suppression with PDE5 Inhibition in End-Stage Multiple Myeloma. Cancer Immunol. Res..

[60] Weed D.T., Vella J.L., Reis I.M., De La Fuente A.C., Gomez C., Sargi Z., Nazarian R., Califano J., Borrello I., Serafini P. (2015). Tadalafil Reduces Myeloid-Derived Suppressor Cells and Regulatory T Cells and Promotes Tumor Immunity in Patients with Head and Neck Squamous Cell Carcinoma. Clin. Cancer Res..

[61] Califano J.A., Khan Z., Noonan K.A., Rudraraju L., Zhang Z., Wang H., Goodman S., Gourin C.G., Marshall L., Fakhry C. (2015). Tadalafil Augments Tumor Specific Immunity in Patients with Head and Neck Squamous Cell Carcinoma. Clin. Cancer Res..

[62] Sevko A., Michels T., Vrohlings M., Umansky L., Beckhove P., Kato M., Shurin G.V., Shurin M., Umansky V. (2013). Antitumor Effect of Paclitaxel Is Mediated by Inhibition of Myeloid-Derived Suppressor Cells and Chronic Inflammation in the Spontaneous Melanoma Model. J. Immunol..

[63] Eriksson E., Wenthe J., Irenaeus S., Loskog A., Ullenhag G. (2016). Gemcitabine reduces MDSCs, tregs and TGFβ-1 while restoring the teff/treg ratio in patients with pancreatic cancer. J. Transl. Med..

[64] Wang Z., Till B., Gao Q. (2017). Chemotherapeutic agent-mediated elimination of myeloid-derived suppressor cells. OncoImmunology.

[65] Vincent J., Mignot G., Chalmin F., Ladoire S., Bruchard M., Chevriaux A., Martin F., Apetoh L., Rébé C., Ghiringhelli F. (2010). 5-Fluorouracil Selectively Kills Tumor-Associated Myeloid-Derived Suppressor Cells Resulting in Enhanced T Cell–Dependent Antitumor Immunity. Cancer Res..

[66] Kanterman J., Sade-Feldman M., Biton M., Ish-Shalom E., Lasry A., Goldshtein A., Hubert A., Baniyash M. (2014). Adverse Immunoregulatory Effects of 5FU and CPT11 Chemotherapy on Myeloid-Derived Suppressor Cells and Colorectal Cancer Outcomes. Cancer Res..

[67] Annels N.E., Shaw V., Gabitass R.F., Billingham L., Corrie P., Eatock M., Valle J., Smith D., Wadsley J., Cunningham D. (2014). The effects of gemcitabine and capecitabine combination chemotherapy and of low-dose adjuvant GM-CSF on the levels of myeloid-derived suppressor cells in patients with advanced pancreatic cancer. Cancer Immunol. Immunother..

[68] Pienta K.J., Machiels J.-P., Schrijvers D., Alekseev B., Shkolnik M., Crabb S.J., Li S., Seetharam S., Puchalski T.A., Takimoto C. (2013). Phase 2 study of carlumab (CNTO 888), a human monoclonal antibody against CC-chemokine ligand 2 (CCL2), in metastatic castration-resistant prostate cancer. Investig. New Drugs.

[69] Nywening T.M., Wang-Gillam A., Sanford D.E., Belt B.A., Panni R.Z., Cusworth B.M., Toriola A., Nieman R.K., Worley L.A., Yano M. (2016). Targeting tumour-associated macrophages with CCR2 inhibition in combination with FOLFIRINOX in patients with borderline resectable and locally advanced pancreatic cancer: A single-centre, open-label, dose-finding, non-randomised, phase 1b trial. Lancet Oncol..

[70] Halama N., Zoernig I., Berthel A., Kahlert C., Klupp F., Suarez-Carmona M., Suetterlin T., Brand K., Krauss J., Lasitschka F. (2016). Tumoral Immune Cell Exploitation in Colorectal Cancer Metastases Can Be Targeted Effectively by Anti-CCR5 Therapy in Cancer Patients. Cancer Cell.

[71] Benner B., Good L., Quiroga D., Schultz T.E., Kassem M., Carson W.E., Cherian M.A., Sardesai S., Wesolowski R. (2020). Pexidartinib, a Novel Small Molecule CSF-1R Inhibitor in Use for Tenosynovial Giant Cell Tumor: A Systematic Review of Pre-Clinical and Clinical Development. Drug Des. Dev. Ther..

